# CASE REPORT Pharyngocutaneous Fistula Closure Using Autologous Fat Grafting

**Published:** 2013-05-09

**Authors:** Geoffrey E. Hespe, Claudia R. Albornoz, Babak J. Mehrara, Dennis Kraus, Evan Matros

**Affiliations:** ^a^Memorial Sloan-Kettering Cancer Center; ^b^New York Head and Neck Institute, New York City, NY

## Abstract

**Objective:** Although the majority of pharyngocutaneous fistulas close spontaneously with conservative measures, 20% to 30% of patients do not respond to this approach, thereby necessitating major reconstruction with adjacent or free tissue transfers. These procedures carry considerable risk, particularly in patients with medical comorbidities or a history of prior surgery/radiation. Less invasive treatment approaches designed to reverse tissue damage or promote spontaneous healing would represent an important medical advance. Autologous fat grafts have been previously shown to promote healing of persistent wounds and improve the quality of radiation-damaged tissue. In this report, successful closure of a persistent pharyngocutaneous fistula with use of autologous fat grafting is described. **Method:** The history and details of pharyngocutaneous fistula closure in a patient with recurrent head and neck cancer are reported. **Result:** A 67-year-old patient with recurrent head and neck cancer and prior radiotherapy underwent reresection including partial pharyngectomy with pectoralis major myocutaneous flap closure. Postoperatively, he developed an enterocutaneous fistula, which failed to close with conservative measures including 8 months of nothing per os. Two rounds of autologous fat grafting were performed with successful fistula healing. **Conclusion:** Autologous fat grafting is a useful treatment for closure of persistent pharyngocutaneous fistulas. Autologous fat grafting should be considered in poor surgical candidates, particularly in the setting of extensive radiation-induced tissue damage.

Standard treatment of pharyngocutaneous fistulas following head and neck surgery is a conservative approach, which includes debridement and drainage until secondary healing occurs. Although spontaneous resolution ensues in the majority of cases, it can be hindered by factors such as radiation, scarring from previous surgery, and malnutrition.^1^ Persistent fistulas require closure with the assistance of vascularized tissue in the form of either a free or local flap.^1^ For a variety of reasons, such as, prior neck vessel exploration, previous flaps, radiotherapy, medical comorbidities, and advanced disease, not all patients are ideal candidates for flap reconstruction. In these situations, less invasive approaches may be considered. Fibrin glue, platelet-derived growth factor and collagen patches have all been reported as successful approaches to promote healing of head and neck fistulas.^2^^-^4 Adipose-derived stem cells (ASCs) extracted from human adipose tissue have been shown to promote healing in chronic wounds.^5^^,^^6^ Autologous fat grafting has been similarly reported to improve radiation-damaged skin; however, these approaches have not been described for repair of persistent pharyngocutaneous fistulas.^7^^,^^8^ In this report, successful closure of a persistent pharyngocutaneous fistula using autologous fat grafting is described.

## CASE REPORT

**Figure 1:**
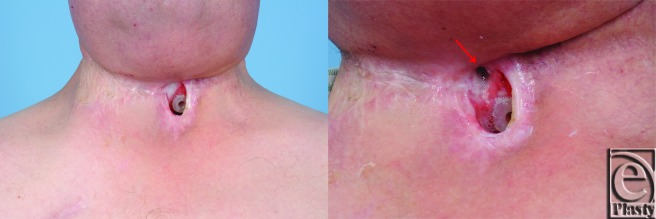
Persistent pharyngocutaneous fistula following 8 months of conservative therapy.

A 67-year-old white man with a history of hypercholesterolemia, morbid obesity, insulin dependent diabetes, and prostate cancer presented in 2007 with a T2N0M0 squamous carcinoma of the left glottic larynx. Treatment was primary chemoradiotherapy. A local recurrence developed in 2009, which was treated with a left type III, modified radical neck dissection, left tracheoesophageal groove dissection, hemithyroidectomy, laryngectomy, and tracheoesophageal puncture. The postoperative course was complicated by a pharyngocutaneous fistula, which resolved with conservative management. In 2011, a regional recurrence of squamous cell carcinoma developed in the right paratracheal groove with overlying skin involvement. A right radical neck dissection, parastomal skin resection, and partial pharyngectomy was performed. Immediate reconstruction consisted of a right pectoralis major myocutaneous flap to close the hypopharynx defect with split-thickness skin grafting externally. The postoperative course was complicated by a pharyngocutaneous fistula at the superior margin of the permanent tracheostoma (Fig 1). Reirradiation was performed perioperatively. Following 8 months of conservative treatment, the patient requested closure of the persistent fistula.

**Figure 2:**
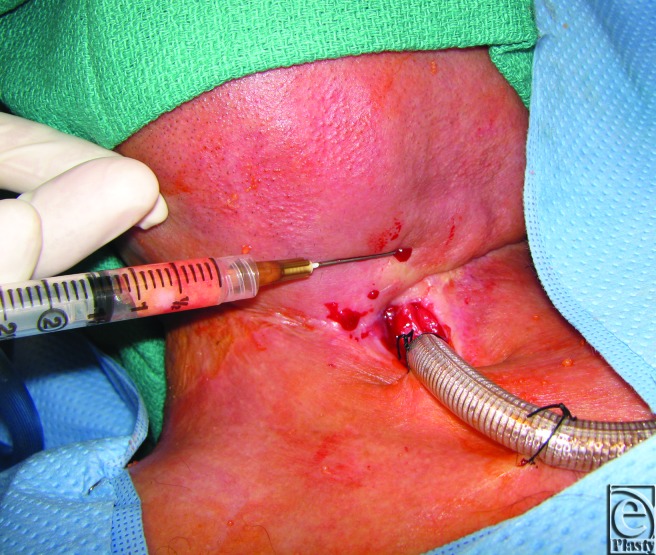
Intraoperative fat grafting to fistula margin.

**Figure 3:**
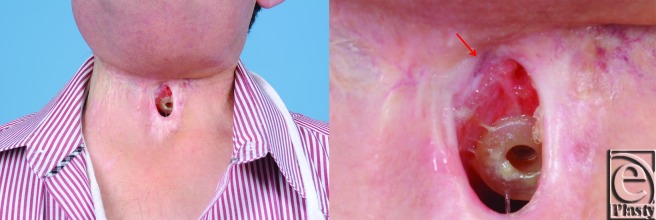
Size reduction of pharyngocutaneous fistula 3 months following first fat grafting.

**Figure 4:**
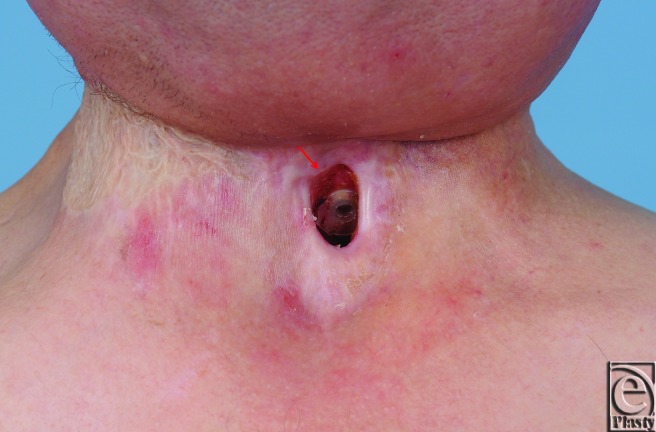
Complete fistula closure 4 months after first fat grafting (includes a second procedure of fat grafting).

Based on previous reports demonstrating recovery of radiated tissue with autologous fat grafting, fat transfer was performed in the current patient to improve tissue quality in preparation for flap transfer or as a primary means of achieving closure. Using hand-assisted liposuction techniques, autologous fat was harvested from the patient's abdomen and purified using Telfa rolling. Approximately 8 mL of purified fat were injected into the area immediately surrounding the pharyngocutaneous fistula using both blunt cannulas and 18 gauge needles (Fig 2).^9^ The tissues were markedly fibrotic; however, use of the sharp needle was helpful in fat transfer. After 3 months of conservative measures (ongoing gastrostomy tube feeds and local wound care), the fistula size reduced by approximately 70% (Fig 3). The quality of the tissues surrounding the fistula improved becoming softer and less fibrotic. Encouraged by these results, a second fat grafting procedure was performed. The edges of the wound were debrided to remove the epithelialized tract and approximately 6 cc of purified lipoaspirate was injected in the tissues surrounding the defect using blunt and sharp needles. One month following the second procedure, the fistula closed spontaneously. It has remained closed 1 year later with the patient tolerating a regular diet (Fig 4).

## DISCUSSION

Pharyngocutaneous fistulas are a major complication following laryngectomy, reported to occur in 3% to 65% of cases.^10^^,^^11^ A conservative approach to the treatment of pharyngocutaneous fistulas will result in eventual closure in the majority of cases; however, a small number of these will remain patent. Persistent fistulas are akin to chronic wounds, sharing many similar characteristics including bacterial colonization, persistent drainage, and chronic inflammation.^12^ At the cellular level, enzymes such as matrix metalloproteinases are elevated in both chronic wounds and distal enteric fistulas.^13^^,^^14^ Arrested healing is present in both pharyngocutaneous fistulas and chronic wounds due in part to impaired angiogenesis, decreased cellular proliferation, reduced growth factor production, and decreased recruitment of endothelial progenitor cells.^15^ Healing is further diminished in cases of prior radiotherapy. Radiotherapy-induced damage begins as injury to the dermal layers of skin, erythema and swelling with progression to ischemia, and finally fibrosis of subcutaneous tissue.^16^ At the cellular level, radiotherapy has been shown to markedly impair resident stem cell function and turnover as a consequence of cell cycle arrest, cellular senescence, and impaired differentiation/proliferation potential.^17^

Studies support that human adipose tissue contains a mesenchymal stem cell population, which may contain regenerative potential.^18^^,^^19^ Introduction of a population of ASCs via fat grafting may be a mechanism to reestablish a favorable wound-healing environment. These stem cells have been previously shown to contribute to tissue repair by expressing cytokines such as vascular endothelial growth factor, insulin-derived growth factor, hepatocyte growth factor, and keratinocyte growth factor.^6^^,^^16^,^18^^-^21 In addition, but to a lesser extent, transferred ASCs have been shown to become incorporated in the local tissues by differentiating into mature tissue cells.^22^ Production of certain collagen types, antioxidants, and cytokines by ASCs can also stimulate healing of damaged tissues.^6^^,^^23^

Greater understanding of ASC biology has led to increased clinical experience with their use both as an ASC isolate and through fat grafting for wound repair. Adipose-derived stem cell injection has led to improved quality and function of damaged tissues in the setting of long-term radiation injury.^16^^,^^24^ Transplantation of autologous fat grafts into the head, neck, and trunk regions in the setting of prior radiotherapy has shown similar results.^7^ Tissue regeneration through introduction of an ASC isolate has been demonstrated in cases of osteonecrosis of the femoral head and meniscal cartilage damage in osteoarthritis.^25^ Extracted ASCs have also been used successfully in the treatment of Crohn's fistulas and a tracheomediastinal fistula caused by laser damage.^26^^-^28 Although the use of isolated ASCs from lipoaspirate differs from fat injection, the aforementioned studies suggest that fat injection is an effective treatment option due to the ASC population contained within.

The current case report suggests that the use of lipoaspirate transfer may be successful in promoting healing of pharyngocutaneous fistulas. Surgical repair of these problems is difficult necessitating procedures associated with considerable risk and failure; therefore, in complex clinical scenarios fat grafting may be a less invasive and safe treatment option to consider prior to proceeding with further surgery. The complete mechanism of healing in this case is not fully understood although it can be hypothesized that introduction of ASCs via fat grafting is a potential method to restore factors missing for proper wound healing. To better understand the mechanism behind healing using fat grafting, more clinical and basic science experience is needed to fully clarify the beneficial effects and safety of ASCs.
